# A Preliminary Study on Sinus Fungus Ball with MicroCT and X-Ray Fluorescence Technique

**DOI:** 10.1371/journal.pone.0148515

**Published:** 2016-03-15

**Authors:** Zidong Jiang, Kai Zhang, Wanxia Huang, Qingxi Yuan

**Affiliations:** 1 Department of Otolaryngology, Peking Union Medical College Hospital, Beijing, China; 2 Institute of High Energy Physics, Chinese Academy of Science, Beijing, China; University at Buffalo, SUNY, UNITED STATES

## Abstract

**Background:**

Sinus fungus ball, an accumulation of fungal dense concretions, is a common disease in practice, and might cause fatal complications or lead to death once converted into invasive type. Early preoperative diagnosis of this disease can lead to appropriate treatment for patients and prevent multiple surgical procedures. Up to now, the diagnostic criteria of sinus fungus ball have been defined and computed tomography (CT) scan was considered as a valuable preoperative diagnostic tool. However, the sensitivity of clinical CT is only about 62%. Thus, investigating the factors which influence sensitivity is necessary for clinical CT to be a more valuable preoperative diagnosis tool. Furthermore, CT scan usually presents micro-calcifications or spots with metallic density in sinus fungus ball. Previous literatures show that there are some metallic elements such as calcium and zinc in fungus ball, and they concluded that endodontic treatment has a strong correlation with the development of maxillary sinus fungus ball and zinc ion was an exogenous risk factor. But the pathogenesis of sinus fungus ball still remains unclear because fungus ball can also develop in other non-maxillary sinuses or the maxillary sinus without root canal treatment. Is zinc ion the endogenous factor? Study on this point might be also helpful for investigating the pathogenesis of sinus fungus ball. In this paper, we tried to investigate the factors which influence the sensitivity of clinical CT by imaging sinus fungus ball with microCT. The origin of zinc ion was also studied through elements test for different fungal ball samples using x-ray fluorescence technique.

**Methods:**

Specimens including fungal ball material and sinus mucosa from patients confirmed by pathological findings were extracted after surgery. All fungal ball specimens came from sphenoid sinus, ethmoidal sinus and maxillary sinus with or without previous endodontic treatment respectively. All of them were imaged by microCT with spatial resolution up to 5μm to acquire three-dimensional structure, and then the heavy metal elements were detected with x-ray fluorescence spectrometer analysis.

**Result:**

High concentration of zinc and calcium were detected in all fungal ball specimens compared to sinus mucosa membrane. Particles with different size varied from disperse to density, which have similar shape to the result of clinical CT but with different size, were found in three-dimensional reconstruction results of microCT.

**Conclusions:**

Spatial resolution is an influent factor of clinical CT sensitivity for sinus fungus ball. Improving the resolution of clinical CT will help to improve its sensitivity. Besides iatrogenic endodontic materials, endogenous metal elements of zinc and calcium might associate with the growth of fungal ball and the micro-calcifications or spots with metallic density of CT imaging.

## Introduction

As a common pathology entity of paranasal sinuses, fungal rhinosinusitis is caused by mycotic infection of nasal cavity and sinus [1–6]. Fungal rhinosinusitis can be generally classified as the invasive and the non-invasive forms [2]. The former is a very serious condition, which leads to death in 90% cases for its fatal intracranial complications [7]. The latter is chronic course, which occurs in immunocompetent patients and can be converted into invasive fungal sinusitis on occurrence of immunocompromised condition of the individual [4]. Due to the development of radiographic imaging technique and the increase in conditions that favor mycotic infections, the number of confirmed cases of fungal rhinosinusitis infection keeps rising in the recent years [4, 8].

Sinus fungus ball, an accumulation of non-invasive fungal dense concretions at the level of the paranasal cavities, was first described by Mackenzie in 1894 [9]. Various terms such as mycetoma, aspergillosis and aspergilloma had been referred to sinus fungus ball as analogous term in the previous literatures [8, 10]. As a saprophytic mold of the ascomycetes class and the dominant fungal pathogen of sinus fungus ball [11, 12], Aspergillus is widely distributed in the environment and can be found in soil, cereals, and decaying vegetation [11]. Aspergillus spores might be inhaled with breathing and stay on mucosa membrane of nasal cavity and paranasal sinuses, which are unlikely to cause disease if the individual is in healthy condition. However, it can be a pathogen and form fungus ball when body resistance drops. Untreated fungus ball of the sinus may occasionally lead to complications [13]. Thus, prompt diagnosis and appropriate initial therapy are essential to avoid a protracted or fatal outcome.

Due to lack of non-specific clinical presentation, most patients are suspected as sinus fungus ball only in case of difficult to treat or recurrent unilateral sinusitis [10, 11]. In such situations, computed tomography (CT) scan becomes the necessary imaging choice. The specificity of CT examination, which relies on its representation of the complete or partial opacification of affected sinus associated with flocculent calcifications [14,15], gives the value close to that of pathological examination [9]. The sensitivity, also called the true positive rate, statistically measures the proportion of positives that are correctly identified as such [16]. For CT examination of sinus fungus ball, the sensitivity relies on its ability to detect characteristic of sinus fungus ball with micro-calcifications or spots with metallic density. The diagnostic criteria based on CT have been clearly defined by deShazo et al. [5] for sinus fungus ball, but Dhong et al. [1] concludes that the sensitivity of CT, in the presence of these findings was calculated about 62%. Prompt preoperative diagnosis, which can lead to appropriate treatment and prevent multiple surgical procedures, is important for patients. Thus, investigating the factors which influence sensitivity is necessary for clinical CT to be a more valuable preoperative diagnosis tool. Assuming that the sensitivity can be increased with the resolution ability improvement of CT device, we tried to image the fungal ball specimens using microCT with higher resolution to investigate this point.

Although sinus fungus ball has been described for more than 120 years, its pathogenesis still remains unclear [3]. Mensi et al. [17] found the incidence of maxillary fungus ball in patients with endodontic treatment was 14 times higher than that of patients in control. Nicolal et al. [18] found the similar metal concentration in the endodontic materials and fungus ball, and suggested that zinc oxide endodontic materials was a risk factor in the pathogenesis of fungus ball. Other literatures [13,19,20] also showed that previous root canal treatment had a strong correlation with the development of sinus fungus ball and zinc ion was exogenous factor. However, some researchers were suspicious of this iatrogenic theory, since it does not explain the cases of affected maxillary sinus without previous root canal treatment and the sinus fungus ball arising in other sinus cavities such as the frontal, ethmoid and sphenoid [3]. Other endogenous factors might play a role in etiopathogenesis of fungus ball and should be taken into account.

In this contribution, we visualized three-dimensional structure of sinus fungus ball using microCT with spatial resolution up to 5μm to reestimate CT procedure in diagnosis of sinus fungus ball. And the X-ray fluorescence spectrometer (XFS), a rapid and nondestructive screening technology for detecting trace elements and heavy metals, was used to determine the origin of zinc ion which is considered relative to the developing of sinus fungus ball.

## Materials and Methods

### Specimen Collection and Preparation

The ethics committee of Peking Union Medical College Hospital granted approval for this study, and written informed consent was obtained from all patients. Specimens were obtained by standard functional endoscopic sinus surgery (FESS) [21] from January 12, 2012 to December 14, 2013, under general anaesthesia in fourteen patients who were histologically confirmed as sinus fungus ball at our hospital. All associated maxillary sinus, sphenoidal sinus, ethmoidal sinus had been treated exclusively by FESS to carry on a wide opening of the affected sinus, allowing a careful extraction of all fungal materials, in the meantime with removal of small piece of the sinus mucous membrane (see Video A in S1 File, https://figshare.com/s/8ca4e387de74cbb35da9). All the samples were then kept in 10% buffered formalin solution for further study. No complication occurred and no medical treatment (antibiotic, antifungal) was required and no recurrence of lesions occurred during one year follow up.

### Histopathologic examination

All of the surgical specimens including fungal ball material and part of sinus mucosa membrane were fixed in 10% buffered formalin and part of each sample were taken and embedded in paraffin. The sections of 6 μm thick was cut and stained with hematoxylin-eosin for histopathologic examination.

### MicroCT imaging and post-processing

All imaging measurements and reconstructions were performed at x-ray imaging station of Beijing Synchrotron Radiation Facility using the MicroXCT-200 system (Carl Zeiss X-ray Microscopy, Inc.). The basic principles of this system are similar to those used in medical CT-scanners. However, recent improvements of X-ray sources and X-ray detector (e.g., charge coupled device (CCD) detectors) make it possible to achieve a resolution down to a few microns. The microCT imaging were performed with the x-ray generator operating at 40kVp and a current of 200 μA, and 2D X-ray projection images (see Fig A in S1 File, https://figshare.com/s/3c92cbc25cac7320af37) are acquired from 900 angles over 180°, resulting a total scanning time of about 3 hours.

Three-dimensional image are reconstructed by applying the “filtered back-projection algorithm for fan-beam geometries” which combines all angular information for every line in the camera to generate the cross-section of the specimen. With this method we reconstructed up to 1024 cross-sections (slice image) of each specimen (see Fig B in S1 File, https://figshare.com/s/f4a756cf14e96b90cb8a), with a lateral resolution and a slice-to-slice distance down to a few microns. Finally, the image post-processing was done with the Avizo 7.0 software package on a Xeon 2660 MHz computer. All the cross-sections were recombined into 3D model of the specimen. The image segmentation was also done based on gray scale to show different structure of the specimen. Since the imaging data only represent absorption values, it is possible to apply “false” color tables to the dataset to produce grayscale or color images and to change the opacity of selected colors or absorption values.

### X-ray fluorescence test

The x-ray fluorescence tests were performed using EAGLE III XXL (EDAX Inc.) fluorescence spectrometer with its x-ray generator operating at 40kVp and a current of 100μA. All the specimens including fungal ball material and sinus mucosa membrane were tested under condition of air to get qualitative elemental information.

## Results

**Fig 1:**
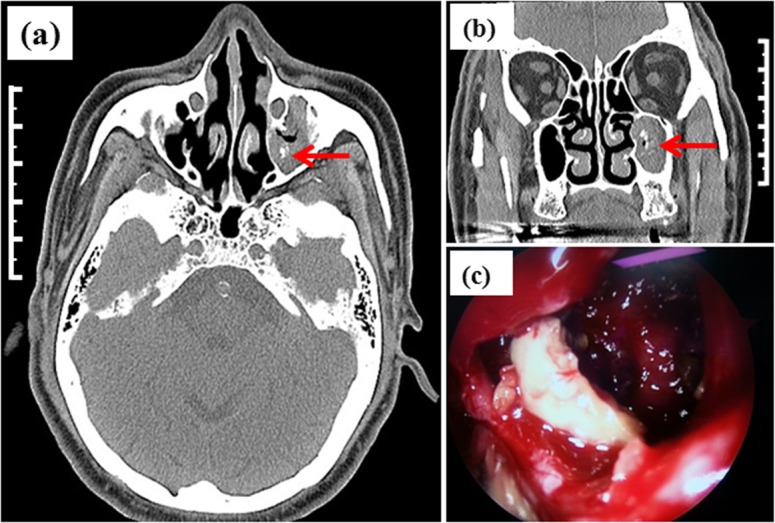
**The axial (a) and coronal (b) CT image of case No.3 and the photograph of fungus ball taken through an endoscope (c)** (The total length of scale bar shown in (a) and (b) is 5 cm.).

Fourteen cases of sinus fungus balls (details in Table 1) were totally examined. The specimens came from six males and eight females, with the ages ranged from 28 to 69 years. All samples of patients with sinus fungus ball were extracted after FESS and confirmed by histopathological results. Three specimens came from the sphenoid sinus, three from ethmoidal sinus and the other eight from maxillary sinus with or without endodontic treatment including two cases affected unilateral maxillary sinus and ipsilateral ethmoid. Histopathological examination proved to be positive for all 14 cases. Preoperative CT scan findings of micro-calcifications or spots with metallic density in heterogeneous opacification sinus cavity are highly suggestive of a fungus ball. Fig 1 shows the axial and coronal CT image of case No.3 and the photograph of fungus ball taken through an endoscope. From Fig 1B, we can clearly see the affected sinus (indicated by the red arrow).

**Table 1:** The detail of fourteen sinus fungus ball cases and specimen.

Case & Specimen No.	Age	Gender	Sinus cavity	Root canal treatment
**1**	**65**	**F**	**S**	**N**
**2**	**69**	**M**	**M,E**	**N**
**3**	**28**	**F**	**M**	**N**
**4**	**35**	**M**	**M,E**	**Y**
**5**	**45**	**F**	**S**	**N**
**6**	**50**	**F**	**M**	**Y**
**7**	**32**	**F**	**E**	**N**
**8**	**28**	**F**	**M**	**N**
**9**	**60**	**M**	**M**	**Y**
**10**	**43**	**M**	**M**	**N**
**11**	**55**	**F**	**E**	**N**
**12**	**56**	**M**	**S**	**N**
**13**	**61**	**M**	**E**	**N**
**14**	**37**	**F**	**M**	**Y**

(F: female; M: male; S: sphenoid sinus; M: maxillary sinus; E: ethmoid sinus; N: nil; Y: yes)

**Fig 2:**
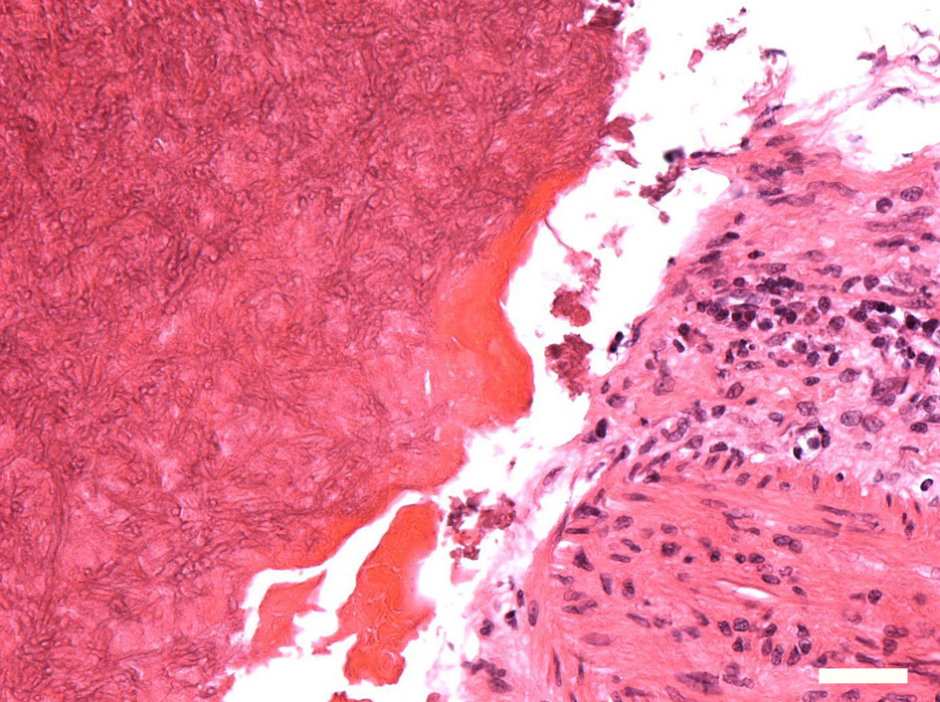
The microscopic evaluation shows the presence of characteristic broad, aspergillus hyphae and a large of chronic inflammatory cell. The scale bar is 50 microns. (Hematoxylin-eosin stain, original magnification ×200.).

Histopathologic evaluation of the sinus fungus ball from all 14 patients revealed similar results. Fungus ball appears as a mass within the lumen of a paranasal sinus and is usually limited to affected sinus as mucopurulent, friable cheesy or clay like material. Within the specimens there was abundant necrotic debris mixed with dense area of broad, septate with 45° dichotomous branching, characteristic of aspergillus and a large of chronic inflammatory cell. Hyphae could not be found within all mucosa samples. Fig 2 gives the microscopic result of 6 μm thick sections of specimen No.3, showing the presence of characteristic broad, aspergillus hyphae and a large of chronic inflammatory cell.

**Fig 3:**
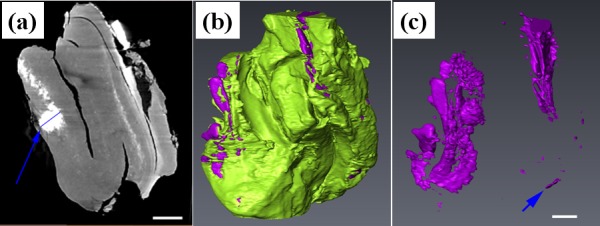
**The microCT coronal image (a), 3D reconstruction result (b) and segmented micro-calcifications and spots with metallic density (c) of No. 3 specimen.** The scale bar shown in (a) and (c) is 500 microns.

Fig 3(A), 3(B) and 3(C) show the microCT coronal image, 3D reconstruction result and segmented micro-calcifications and spots with metallic density respectively of No.3 specimen. From Fig 3(A), it can be clearly seen that differentiates individual segments of the fungus ball, namely calcification, and the calcification is composed of numerous tiny particles. The length indicated by blue line in Fig 3(A) is about 450 microns, which can be seen in the clinical CT image. In Fig 3(C), the tip size of a calcification part indicated by the arrow is about 20 microns, and some spots whose size is around 10 microns can be seen. From Fig 3, we can see that the particle whose size is about 10 microns can be clearly seen using microCT.

**Fig 4:**
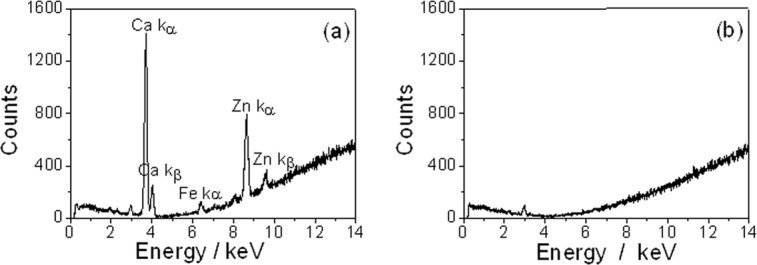
Fluorescence spectrum of specimen No.3 (a) and mucosa (b) show high concentration of Ca and Zn elements in the specimen No.3.

X-ray fluorescence technique offers elemental analysis with high sensitivity, which allows the role of trace elements to be investigated in specimen. From the x-ray fluorescence results, we can obviously see K_α_ and K_β_ emission line of calcium and zinc in all fourteen fungus ball specimens, however almost none in sinus mucosa. A typical fluorescence spectrum of sinus fungus ball (specimen No.3) was shown in Fig 4(A) (from Data A in S1 File, https://figshare.com/s/46689b2d4a38551b8e29), and the fluorescence spectrum of mucosa was shown in Fig 4(B) (from Data B in S1 File, https://figshare.com/s/c29bb49284ac8486fe92). We can see that K series emission line of Ca and Zn are very strong in Fig 4(A), qualitatively showing relatively high concentration of those two elements in the specimens. Because all specimens were tested under the condition of air, the Compton scattering of air is unavoidable, resulting in the base line of spectra ascending with energy.

## Discussion

Histopathologic finding is the golden standard for fungus ball diagnosis postoperative [10]. However, early preoperative diagnosis of sinus fungus ball can prevent multiple surgical procedure and lead to effective treatment for patients. With the preoperative assessment with clinical CT imaging, surgical plan will be well prepared including surgical extent and position. One of the main clinical criterion to diagnose a sinus fungus ball is radiological opacification evidence of affected sinus with or without associated flocculent calcifications in the lumen of sinus [5]. Because the spatial resolution of currently used clinical CT is about 300 microns, particles with its size smaller than 300 microns will not be visualized by clinical CT. We assume that the sensitivity can be increased if the resolution of CT image is improved. In our study, we found mottled hyper dense foci of variable size in the center of homogenous opacification, which are the consequence of the high content of heavy metals deposition. According to microCT results (Fig 3), we can not only clearly see the particles with size around 10 microns, but also can see the similar shape of micro calcifications or spots with metallic density. Moreover, all cases were histologically confirmed as sinus fungus ball. Thus, we concluded that the diagnostic accuracy of CT examination will be improved if its resolution ability is improved in the future, resulting in higher sensitivity and a more valuable tool for preoperative diagnosis.

The general CT findings are micro-calcifications or spots with metallic density in sinus fungus ball. Previous literatures [1,5,11] show that there are some mental elements such as calcium and zinc in fungus ball and they concluded that those metal elements had a strong correlation with the development of sinus fungus ball. But the origin of those elements is not confirmed. Previous work [17, 18] suggested that zinc oxide endodontic materials were an exogenous risk factor. Some researchers think that functional blockage of the sinus ostium had been conjectured as a mechanism of pathogenesis of sinus fungus ball [22]. But Tung-Lung Tsai found that there is no relationship between obstruction of the sinus ostium and sinus fungus ball [23].

Generally, heavy metals and trace elements in materials can be determined with several methods such as atomic absorption spectrometry (AAS), inductively coupled plasma mass spectrometry (ICP-MS), inductively coupled plasma optical emission spectrometer (ICP-OES) after acid digestion of the specimens[24, 25]. All these three methods are time consuming and might cause the inputs of cross contamination among samples. The x-ray fluorescence (XRF) technique is a novel and non-destructive instantaneous method in measuring the concentration of multi-elements. Compared with ICP-MS and ICP-OES, XRF doesn’t need complicated sample preparation. Moreover, because of the non-destructive property of XRF, the sample can be kept and used for following histopathologic examination after XRF test. According to our XRF results, we can obviously see K_α_ and K_β_ emission line of calcium and zinc in all fungal ball specimens, and almost none from sinus mucosa. Those results cannot support the assumption that zinc ion in fungus ball come from endodontic materials. We concluded zinc might be an endogenic factor. In fact, a number of heavy metallic elements, including copper, zinc, iron, calcium and so on, are essential for fungus growth and metabolism activation. As metallic elements, zinc and calcium will also contribute to micro calcifications or spots with metallic density of CT image.

FESS is the gold standard for treatment of sinus fungus ball without necessary following antifungal therapy [26, 27]. However, some researchers found it is impossible to visualize all angle due to the blockage from the bone of lachrymal ducts, and FESS can leave fungal particle component in affected maxillary sinus after surgery, resulting in about 5% relapses ratio [28]. According to the XRF results, the Ca and Zn elements were found in all fungal ball specimens, meaning that those elements might be necessary for fungus growth. This finding will not only help to develop flushing fluid for postoperative cavity washing to inhibit fungus growth and reduce recurrence, but also help to explore the pathogenesis mechanism of sinus fungus ball and evaluate whether the adoption of preventive measurements can reduce the occurrence of sinus fungus ball.

The study in this contribution is limited. Next step, we will study those microCT imaging features of the sinus fungus ball in a larger number of patients and especially of those patients, whose clinical CT results showing no micro-calcifications or spots with metallic density. Studies will be in bulk sample, multicenter and controlled studies to determine the calcium ion and zinc ion and other heavy metal ions in sinus fungus ball. These findings might be able to strongly emphasize the crucial role of CT scan in the early diagnosis of fungal sinusitis in future and discover the mystery issue why the fungus ball mostly involved unilateral nasal sinuses.

## Supporting Information

S1 File Data A, Fluorescence data of No.3 specimen acquired by EAGLE III XXL (EDAX Inc.). Channel range “0 to 4000” of the data is equivalent to “0 to 40 keV”. https://figshare.com/s/46689b2d4a38551b8e29. Data B, Fluorescence data of mucosa acquired by EAGLE III XXL (EDAX Inc.). Channel range “0 to 4000” of the data is equivalent to “0 to 40 keV”. https://figshare.com/s/c29bb49284ac8486fe92. Fig A, 2D projection images acquired by microCT of specimen No. 3. All 900 projection images were acquired at 900 rotation angular positions from -90° to 90°. https://figshare.com/s/3c92cbc25cac7320af37. Fig B, Reconstructed slice images from all projection images acquired by micro CT. https://figshare.com/s/f4a756cf14e96b90cb8a. Video A, The video of specimen extraction process by standard functional endoscopic sinus surgery (FESS). https://figshare.com/s/8ca4e387de74cbb35da9.
(DOCX)
