# Supplementary material for: A Preliminary Study on Sinus Fungus Ball with MicroCT and X-Ray Fluorescence Technique
Source: PLoS One. 2016 Mar 15;11(3):e0148515. doi: 10.1371/journal.pone.0148515 (PMC4792473; doi:10.1371/journal.pone.0148515)
Supplement: S1 File — Data A, Fluorescence data of No.3 specimen acquired by EAGLE III XXL (EDAX Inc.). Channel range “0 to 4000” of the data is equivalent to “0 to 40 keV”. https://figshare.com/s/46689b2d4a38551b8e29. Data B, Fluorescence data of mucosa acquired by EAGLE III XXL (EDAX Inc.). Channel range “0 to 4000” of the data is equivalent to “0 to 40 keV”. https://figshare.com/s/c29bb49284ac8486fe92. Fig A, 2D projection images acquired by microCT of specimen No. 3. All 900 projection images were acquired at 900 rotation angular positions from -90° to 90°. https://figshare.com/s/3c92cbc25cac7320af37. Fig B, Reconstructed slice images from all projection images acquired by micro CT. https://figshare.com/s/f4a756cf14e96b90cb8a. Video A, The video of specimen extraction process by standard functional endoscopic sinus surgery (FESS). https://figshare.com/s/8ca4e387de74cbb35da9. (DOCX) [file pone.0148515.s001.docx]

**Data A. Fluorescence data of No.3 specimen acquired by EAGLE III XXL (EDAX Inc.).** Channel range “0 to 4000” of the data is equivalent to “0 to 40 keV”.

[https://figshare.com/s/46689b2d4a38551b8e29](https://figshare.com/s/46689b2d4a38551b8e29)

**Data B. Fluorescence data of mucosa acquired by EAGLE III XXL (EDAX Inc.).** Channel range “0 to 4000” of the data is equivalent to “0 to 40 keV”.

[https://figshare.com/s/c29bb49284ac8486fe92](https://figshare.com/s/c29bb49284ac8486fe92)

**Fig A. 2D projection images acquired by microCT of specimen No. 3.** All 900 projection images were acquired at 900 rotation angular positions from -90° to 90°.

[https://figshare.com/s/3c92cbc25cac7320af37](https://figshare.com/s/3c92cbc25cac7320af37)

**Fig B. Reconstructed slice images from all projection images acquired by micro CT.**

[https://figshare.com/s/f4a756cf14e96b90cb8a](https://figshare.com/s/f4a756cf14e96b90cb8a)

**Video A. The video of specimen extraction process by standard functional endoscopic sinus surgery (FESS).**

[https://figshare.com/s/8ca4e387de74cbb35da9](https://figshare.com/s/8ca4e387de74cbb35da9)
